# Mass spectrometry-based proteomics in Chest Medicine, Gerontology, and Nephrology: subgroups omics for personalized medicine

**DOI:** 10.7603/s40681-014-0025-y

**Published:** 2014-11-13

**Authors:** Shih-Yi Lin, Wu-Huei Hsu, Cheng-Chieh Lin, Chao-Jung Chen

**Affiliations:** 1 Institute of Clinical Medical Science, China Medical University College of Medicine, 404 Taichung, Taiwan; 2 Department of Internal Medicine, China Medical University Hospital, 404 Taichung, Taiwan; 3 Division of Nephrology and Kidney Institute, China Medical University Hospital, 404 Taichung, Taiwan; 4 Division of Pulmonary and Critical Care Medicine, China Medical University Hospital and China Medical University, 404 Taichung, Taiwan; 5 Department of Family Medicine, China Medical University Hospital, 404 Taichung, Taiwan; 6 Graduate Institute of Integrated Medicine, College of Chinese Medicine, China Medical University, No. 91, Hsueh-Shih Road, 402 Taichung, Taiwan; 7 Proteomics Core Laboratory, Department of Medical Research, China Medical University Hospital, 404 Taichung, Taiwan; 8 School of Medicine, College of Medicine China Medical University, No. 91, Hsueh Shih Road, 404 Taichung, Taiwan

**Keywords:** Mass spectrometry;, Personalized medicine;, Proteomics;, Chest Medicine;, Nephrology;, Gerontology

## Abstract

Mass spectrometry (MS) is currently the most promising tool for studying proteomics to investigate largescale proteins in a specific proteome. Emerging MS-based proteomics is widely applied to decipher complex proteome for discovering potential biomarkers. Given its growing usage in clinical medicine for biomarker discovery to predict, diagnose and confer prognosis, MS-based proteomics can benefit study of personalized medicine. In this review we introduce some fundamental MS theory and MS-based quantitative proteomic approaches as well as several representative clinical MS-based proteomics issues in Chest Medicine, Gerontology, and Nephrology.

## 1. Mass spectrometry and proteomics

Proteomics (large-scale analysis of proteins) can directly reflect and characterize the biological function, pathways, activities and subcellular distributions, and thus is most promising and applicable in biomedicine [1]. Mass spectrometry (MS) has become a mainstream and dominant analytic tool for studying proteomics due to high sensitivity, specificity and high throughput in protein characterization including posttranslational modifications [2,3]. Given powerful technology to decipher biological processes, ever more investigators apply MS-based proteomics to clinical research. This review provides an uncomplicated but broad overview of background and issues in MS-based proteomics: protein digestion, instrumentation, ionization methods, database search, quantitative proteomics. We also discuss MS-based proteomic strategy applied in Chest Medicine, Gerontology, and Nephrology.

### 1.1. Sample preparation: gel- and solution-based digestion

For identification, proteins can be analyzed with intact form for top-down analysis or enzymatically into peptides for bottom up analysis. Since MS techniques are more sensitive for peptides than for proteins, most proteomic applications adopt bottom-up analysis; enzymatic (such as trypsin) digestion, is widely used to digest proteins into peptides in gels or in solution prior to MS analysis. Gel-based digestion is often used when complex proteins are separated on one- or two-dimensional gel electrophoresis. After separation, proteins trapped in gel spots are excised, washed, then digested with trypsin *in situ*. Digested peptides were often extracted from gel pieces with sequential extraction of 0.1% formic acid (FA), 50%ACN/0.1%FA and pure ACN. Because urea, detergents (SDS, Triton X-100) and salts greatly reduce analyte signals in ESI-MS and MALDI-MS while impairing LC separation, removal of contaminants is a key step in sample preparation [4]. One advantage of gel-based digestion: surfactants and salt contaminants are expunged from gels by washing steps without significant protein loss [5, 6]. Practical gel-assisted digestion for surfactant-enriched protein sample preparation starkly increased membrane proteome recovery [7]. Still, digested peptide recovery of gel-based digestion is often limited by lower extraction efficiency of trapped peptides from gel spots; excised gel spots must be cut into smaller pieces for better digestion and extraction efficiency.

In solution-base digestion, urea, detergent or heat is usually added to denature protein for efficient enzymatic digestion. Without trapping proteins in gel, solution-based digestion benefits from higher peptide recovery. However, salts, urea and detergents for digestion must be removed by solid phase extraction (C18 stationary phase) before MS analysis. Recently, a simple universal sample preparation by a filter-aided method developed by M. Mann [8] allowed researchers to use higher amount of detergent or urea for comprehensive proteome analysis. Its lone drawback is longer processing time in multiple centrifugations. Trypsin is most commonly used, owing to high cleavage efficiency and specificity in targeting arginine and lysine at C-terminal. Tryptic peptides are primarily of ideal size and multiply charged suited for identification by tandem mass spectrometry (MS/MS) [9, 10]. In analyzing complex proteome, additional enzyme of endoproteinase Lys-C can be used with trypsin to boost protein digestion efficiency by eliminating the majority of missed cleavages.

## 2. Ionization methods of ESI and MALDI

ESI and MALDI are two chief ionization methods for charging and transforming proteins/peptides into gas phase available for MS analysis [11]. ESI, meaning dissipate liquid sample homogeneously, was not applied to analysis of large molecules until 1988. John Fenn *et al*. demonstrated its capacity for analyzing large biomolecules [12]. By applying positive or negative direct current (DC) voltage (+2~4 kV or -2~4 kV) at an electrically conducted spray tip, sample solution is dispersed by electrospray into a fine aerosol. Sprayed fine aerosol were charged and continuously evaporated based on ion evaporation model and charge residue model, which allow analyte charged in gas phase and transferred into MS analyzer [13]. When operating flow rate is above the optimal spray flow rate of the ESI tip orifice, ESI ion signals increase linearly with analyte concentrations until it saturates in MS analyzer system [14]. For more sensitive ESI-MS analysis, Wilm and Mann have introduced nanoelectrospray (nanoESI) technique [15] that uses extremely small needle orifices (nanospray tips with 20 μ orifice iscommercially available) for spray flow rate below 1 μl/min. Initial created smaller droplets enable establishment of high surface-volume ratio of droplets, early fissions without extensive evaporation, thus increasing sampling efficiency and tolerating higher salt contamination. Since nanoESI is operated in nanoliter flow rate, nanoESI is broadly coupled to nanoLC (LC flow rate: 200-400 nl/min) for more sensitive analysis in proteomics [16].

MALDI is a technique involving serial energy transfer and ionization processes. Samples are first mixed with MALDI matrix (i.e. α-Cyano-4-hydroxycinnamic acid (CHCA), 2,5-dihydroxybenzoic acid (DHB), Sinapinic Acid (SA)) on a spot of a MALDI plate. After air-dry and cocrystallization, sample and MALDI matrix are colocolized in crystals. With laser beam irradiation on the crystals, MALDI matrix absorbs laser energy and help analytes desorb from crystals into gas phase [17]. Homogenous crystalscan be observed by video camera set up in MALDI ion source and can provide better signal reproducibility and sensitivity. When applying matrix on samples, the ratio of matrix and analyte sometimes should be optimized for better sensitivity. In addition, thicker crystals significantly reduce peak resolution. Because sample spot homogeneity is the major concern to influence signal reproducibility in MALDI, hydrophobic MALDI target has improved sample homogeneity as well as concentrate analytes [18]. MALDI has been broadly used in analyzing small molecules, polymer, peptides, proteins, oligonucleotide sequencing, and DNA [19]. Compared with ESI ionization method, MALDI has advantages of rapid sample preparation, and more tolerance of salts and detergents. However, because MALDI is usually suffered from poor reproducibility in absolute signal intensity from sample well-to-sample well, MALDI is not commonly used in absolute quantitative approach unless an internal control signal was introduced [20].

## 3. Basic description of mass analyzer

In the growing field of proteomics, some major types of mass analyzers are frequently used, such as triple quadrupole, ion trap, orbitrap, fourier transform ion cyclotron resonance (FT-ICR) and TOF instruments [21]. Each analyzer has its superiority and limitations in performance: e.g., intra-spectrum dynamic range (the range over which the ion signal is linearly proportional to the analyte concentration), sensitivity, mass range, scan speed, scanned duty cycle, accuracy, and resolving power (the ability to differentiate two adjacent peaks). These analyzers can operate alone or couple in series, named hybrid mass spectrometer: e.g., quadrupole-TOF, quadrupole-orbitrap, ion trap-orbitrap, ion trap- TOF, ion trap-FTICR etc., to provide a better performance by merging the strengths of each [22].

**Fig. 1:**
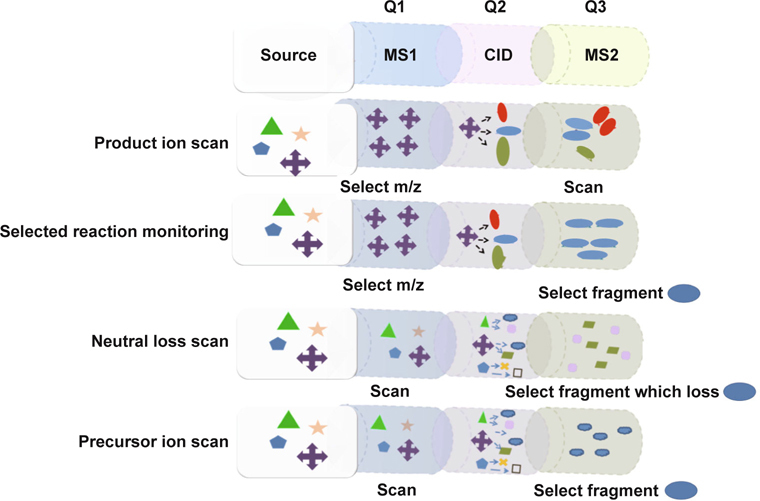
Scan modes of tandem mass spectrometry. (1) Product ion scan: select Q1 precursor ion and scan Q3 production. (2) Selected ion monitoring: select precursor ion in Q1 and monitor one or more fragment ions in Q3. (3) Neutral loss scan: scan all ions in Q1 and select ions with neutral loss in Q3. (4) Precursor ion scan: scan precursor ion in Q1 and select certain fragment ion in Q3, all collision induced dissociation carried out in Q2.

In quadrupole-MS, ion mass scan is carried out by creating time-varying electric fields constructed by DC and RF voltage on four hyperbolic rods positioned symmetrically along one axis. Potential of DC applied to adjacent rods are opposite to each other. Combined DC and RF voltage can then create a stability potential diagram for a given ion mass stably pass through the quadruple and be detected [23]. Thus, quadrupole can act as a mass filter for ion mass scan by varying the RF and DC voltages or as an ion guide for ion transmission ion by setting RF voltage only. In tandem MS of triple quadrupoles, the first quadrupole (Q1) act as a mass filter for ion scan or ion selection, the second quadrupole (Q2) act as ion guide with RF only mode for collision induced dissociation of ions, which were then scanned by the third quadrupole (Q3). Figure 1 shows different scanning modes by MS/MS. This tandem MS (MS/MS) in space includes precursor ion scan, product ion scan, neutral loss scan, selected ion monitoring (SRM), and multiple reaction monitoring (MRM), which can greatly reduce chemical noises to improve sensitivity. MRM, a scan mode of multiple SRM transitions within the same MS analysis, detects precursor/fragment ion pairs. Due to superior sensitivity of MRM function, nanoLC-ESI triple quadruple have been developed for biomarker validation in large sample size in target proteomics instead of ELISA and Western blot [24, 25].

Similar to quadrupole-MS, the operating principle of ion trap is based on electric fields constructed by a ring (RF voltage) and two end caps (alternating current (AC) voltage), which create a stable potential diagram for storage a given mass ion in an ion trap. For scanning ions of an ion trap, ions are detected after they exited the end cap electrode by ramping RF voltage on ring electrode or by causing resonant ejection on end cap electrodes [26]. Advantages of ion trap analyzer include fast scan speed, MS^n^ ability (e.g. MS, MS/MS and MS/MS/MS), and high sensitivity [27].

Ion trap can perform MS/MS in time with product ion scan and MRM. Yet when overloading ions in the ion trap, space-charge effect will result in poor peak resolution and mass-shift. Therefore, most ion trap equipped with pre-analysis function (automatic gain control, AGC) to estimate proper ion loading time [28]. The geometries of electrotrodes have revolutionized from threedimensional to linear ion trap to upgrade efficiency and capacity, sensitivity, detection dynamic range, and scan rate [29].

In TOF analyzer, mass-to-charge (m/z) ratio of each ion is determined by flight time of charged ions over a vacuum tube of specified length inversely proportional to [30]. Because TOF can record all ions simultaneously and separate ion based on each m/z ratio, it is superior of fast scan speed. However, the resolution and mass accuracy of TOF is dampened by several factors including sample thickness difference, initial ion velocities difference, and turn around effects, etc. [31]. Design of delay extraction and reflectron have greatly improved resolving power, mass accuracy, and prompt TOF as high-resolution mass analyzer [32]. Nowadays TOF is frequently coupled with another TOF (such as MALDI TOF-TOF) or quadruple (Q-TOF) for high-quality MS/MS spectrum. Owing to feasibility with LC-ESI system, nanoLC-ESI-Q-TOF has been widely used in bottom-up proteomics for high resolution, high scan speed, and high mass accuracy [33].

In FT-ICR-MS, with applying a homogenous unidirectional magnetic field, ions of specific m/z will undergo cyclotron motion with corresponding frequency characteristic of their m/ z ratio after excited by resonant rf electric field [34]. All ions of the same m/z travel in a spatially coherent packet. Each ion packet induces current on a pair of opposed electrodes to yield time-domain signal then deconvoluted by Fourier transformation to obtain their corresponded m/z [35]. Among current mass analyzers, FT-ICR affords highest mass resolving power (~1,000,000 at FWHM) and mass accuracy (<1 ppm) [36]. High accuracy of measuring mass of analyte ion can help to determine its accurate elemental composition. In proteomics, high resolution power of FT-ICR has superiority in identifying protein either using “bottomup” or “top-down” approach [37, 38]. Still, with scan speed of FT-ICR MS slower, FT-ICR MS is not widely used in quantitative proteomics, yielding less peptide fragmented ion spectra in complex proteome samples [37].

Orbitrap-MS can be viewed as a modified form of Kingdom trap or modified form of ion trap. The difference between orbitrap and ion trap is that the field of orbitrap is electrostatic while the field of quadrupole ion trap is electrodynamic [29]. Advantages of orbitrap include high mass accuracy, and less space-charge effects thus wilder dynamic range and higher high mass/charge range [29]. However, because the mass signals were based on the imaging current, orbitrap is still limited by its slower scan speed compare to ion trap and TOF systems. Linear ion trap triple quadruple (LTQ) and quadruple have been both successfully hybrid to orbitrap by insertion of a c-trap, which can storage ions and reduce kinetic energy of ions from ion source and then injected ions into orbitrap for analysis. In initial development of LTQ-orbitrap design, orbitrap was used for precursor ion scan (MS scan) to obtain accurate ion mass with high resolution, and LTQ was used for product ion scan (MS/MS scan) to obtain abundant MS/MS spectra with high throughput. Parallel scans can dissolve slow scan rate problem of orbitrap in proteomics [39]. Recently, higher energy collision dissociation (HCD) cell was adjacent to orbitrap for performing quadruple-like MS/MS without losing low mass ions. This improved MS/MS function allows LTQ orbitrap-MS applicable to iTRAQ quantitative proteomics, in which low mass ion tags (114, 115, 116, 117 m/z) in MS/MS spectra were used for quantitation. More recently, high field of orbitrap has improved scan speed to 18 Hz at resolution setting of 15,000 at 200 m/z. (Q Exactive HF, Thermo).

## 4. Database searching and protein identification

MS-based approaches are current popular methods for protein identification based on well-established genomic and protein databases as well asbioinformatics tools. Higher mass accuracy and resolution of MS data can provide more confidence protein identifications. The “peptide mass fingerprinting” (PMF) method is the fastest method to identify proteins recovered from 2DE-based proteomics. In PMF, proteins are first digested, then detected by MS full scan to obtain peptide signals as many as possible. Detected peptide signals are compared with theoretically expected peptide masses in a protein database. A score was used to describe results of each comparison [40]. For protein identification with MS/ MS spectra, *de novo* sequencing and “peptide fragment fingerprinting” are widely used. *De novo* methods are used to identify proteins when genomes are not known and utilize computational approach to deduce (partial) sequence of peptides directly from experimental MS/MS spectra [41]. Peptide fragment fingerprint entails comparing experimental MS/MS spectra against those theoretically generated peptide candidates [42]. There are numbers of searching algorithms for protein identification: e.g, probability-based scoring in MASCOT, cross correlation scoring in SEQUEST, and hypergenomic scoring in X!TEM [43-45]. Searching algorithms can only identify proteins with sequences in database, while bottom-up method has limitations: e.g., unanticipated tryptic cleavage by-products, limited identification rates of LC-MS/MS runs [46].

### 4.1. Quantitative proteomics

MS-based quantitation has been a major wok in proteomic research [47]. The current available quantitative methods divide into gel-based and gel-free nano LC-MS/MS quantitative proteomics [48, 49]. In gel-based proteomics, complicated protein mixture was analyzed by two-dimensional gel electrophoresis (2DE). Protein mixture was first separated on an immobilized pH gradient strip according to isoelectical points of proteins. Then, the strip was put on the top of SDS-PAGE for the second dimensional separation according to their molecular weight. 2DE presents a quantitative map of proteome, providing information about the estimated pI and molecular weight of proteins, the levels of protein expression, and post-translation modification [6]. Each individual sample or pooled sample group is performed on each gel. Replicated runs should be performed to reduce quantitative errors. In quantitation, 2D-gels of different sample groups were recorded by image software, which can calibrate spot location and intensity, and output relative quantitative information.

Gel-based approaches have several important advantages for complex protein mixtures: high resolution for separating complex proteins, visualized post-translational modifications and removable salts/detergents in gel-based protein digestion procedure [50, 51]. However, the well-recognized limitations of 2DE include low reproducibility, and inability of analyzing membrane proteins. To improve reproducibility and accuracy in quantitation of 2DE, difference gel electrophoresis (DIGE) approach was introduced by the principle of fluorescence pre-labelling sample proteins before 2D electrophoresis. Proteins of different sample group are separately labeled with different fluorescence reagents (cyanine dyes with different excitation and emission wavelengths) and then pooled into a sample mixture, followed by 2D electrophoresis analysis on a single gel. The different extracted proteins can be visualized under corresponding excitable wavelength and then enable the comparative quantitation among these proteins [48]. However, the colorless DIGE gels should be stained with Coomassie blue or silver stain and carefully recalibrated of spot position before excising interested protein spot for MS analysis.

The other major quantitative proteomic strategy is the gel-free approach using nano LC-MS/MS which can be further divided into reagent labeled and label free approaches.

The label-based technologies are based on the principle that labelled peptides in different sample groups with a combination of non-radiative isotopes (e.g. C^13^, H^2^, N^15^). The different sample groups labeled with different tags were then mixed into the same sample solution prior to nano LC-MS/MS analysis. In nano LCMS/ MS analysis, peptides of same amino sequence from different sample group labeled with different mass tags exhibit the same chromatographic and ionization properties but can be distinguished from each other by a mass-shift signature in MS spectra or mass tags in MS/MS spectra [52]. The label-based technologies include chemical labeling methods of isotope-coded affinity tags (ICAT), isotope-coded protein labelling (ICPL), isobaric tags for relative and absolute quantification (iTRAQ), tandem mass tag (TMT), metabolic labeling SILAC, and ^14^N/^15^N Labelling [53-56]. These labeling techniques contain well-designed isotope reagents to label peptides (ICPL, ICAT, iTRAQ, TMT) or proteins (ICPL, SILAC) for comparing protein expression changes in different biological samples.

Compared with label-based quantitation approaches, label free quantitation has the advantages of lessening the time and complexity of multi-step labeling process, minimizing the sample loss, and the cost of reagents [57]. In label-free methods, each sample or pooled sample group should be separately analyzed without mixing with other sample group. There are two kinds of label-free quantitation approaches based on spectra counting and ion intensity. Spectra counting, defined as by comparing the number of identified MS/MS spectra, is a semi-quantitative approach providing a low cost and rapid evaluation of protein expression difference. Liu *et al*. have observed a strong linear correlation between MS/MS spectra counts and relative protein abundance [58]. Many strategies and statistical tools have been developed for analyzing spectral count data and reducing the variations from replicated runs [59-62]. emPAI (exponentially modified PAI) has been developed to estimate the abundance of proteins [59], which has been incorporated into MASCOT (a commonly-used protein searching engine) for rapid evaluation of protein abundance. However, spectra counting results are only acceptable for samples with relative large quantitative differences and for proteins having the numbered of identified peptides exceeding a certain threshold [63].

Label-free quantification approaches based on ion peak intensities by extracting ion chromatogram (EIC) from MS spectra is more acceptable due to its better quantitation results. In each nanoLC-MS run, the intensity and elution time of each peptide ions was processed, recoded as a quantitative “molecular feature” and form a feature map. These feature ions of different feature maps acquired from different nano LC-MS runs are aligned according to their accurate masses and reproducible LC retention time. Comparison of feature abundances on different maps (representing nano LC-MS runs of different samples) reveals relative changes between peptide amounts [64]. Peptide ions with relative fold changes were then integrated to its MS/MS spectra and protein database search to obtain the protein sequence. Fold changes of all peptides from the same protein are averaged to obtain relative expression level. More concepts and tools for label-free peptide quantification has been recently reviewed by Nahnsen S. *et al*. [65].

**Fig. 2:**
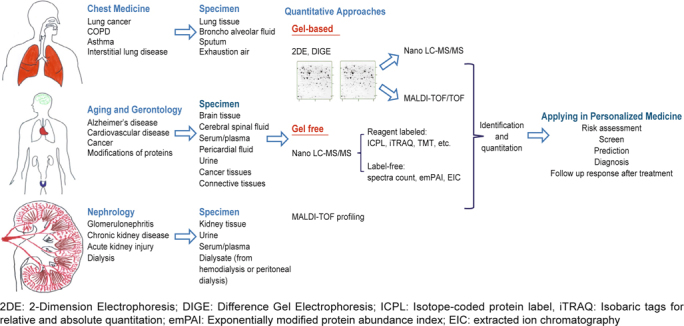
Concept and current progress of mass spectrometry-based proteomics in Chest Medicine, Aging and Gerontology, and Nephrology.

In addition to nano LC-MS/MS approaches, MALDI has also been used as an relative quantitative tool to rapidly discover biomarkers in bacterial [66], serum, urea and saliva. With coating different stationary phases (C18, C8, ion exchange, etc.) on magnetic particles or on MALDI plate (surface-enhanced laser desorption/ionization (SELDI), *et al*. [67]) for specific biomolecular purification, these sample preparation methods can simplify sample complexity and therefore enhance detection sensitivity of certain species. In addition, SELDI-TOF or MALDI-TOF with magnetic particle purification approaches in protein profiling can detect expression changes of protein isoforms. However, these MALDI-TOF based protein profiling methods are still restricted by poor sensitivity in detecting larger proteins (>20 kDa) and ion suppression effects which results in limited peak ions in complex samples.

## 5. MS-based proteomics in Chest Medicine, Gerontology, and Nephrology

The field of MS-based proteomics has getting matured, being able to analyze the complex proteome with consistency, and even to explore proteome dynamics [68]. For years, the gap and transition between discovery science and clinical medicine has been wide and slow. However, the potential for MS-based proteomics, applied as a methodology in clinical practice, is promisingly powerful to bridge the gap and accelerate the transition. Here, we describe applications of MS-based proteomics in Chest Medicine, Gerontology and Nephrology, and the general approaches and workflow was shown in Figure 2.

### 5.1. Chest Medicine

The majorities of proteomic studies in Chest Medicine have been straightforward focusing on major diseases: e.g., lung cancer, obstructive airway diseases like chronic obstructive pulmonary disease (COPD) and asthma. Some MS-based proteomics studies of lung cancer, COPD and asthma were summarized in Table 1 [69- 81]. Lung cancer is the cancer leading high mortality worldwide [82]. The delayed diagnosis at last advanced stage of lung cancer accounts for its high cancer-related death rate. Several studies have identified certain mutation of susceptible genes to lung cancer, including epidermal growth factor receptor (EGFR) gene and nucleotide excision repair genes [83, 84]. Smoking, radon, secondhand tobacco smoke, and other indoor air pollutant are wellrecognized environmental carcinogens of lung cancer [85]. Since the pathogenesis of lung cancer involves the complex interaction of host genetic predisposition and environment, it is unlikely to diagnose lung cancer based on the incomplete picture provided by gene profiling and exposed environmental factors of individuals [86]. Several screening tools as sputum cytology, interval chest x-rays, and computed tomography scans in smokers have proven cost-ineffective in reducing lung cancer mortality rates [87].

MS-based proteomics strategy has shown potential in finding out the biomarkers of lung cancers from several perspectives inclusive of proteome of lung cancer tissue, serum, saliva, braonchoalveolar fluid, and exhaustive air [69-73]. Protein profiles of tissue can distinguish lung tumor from normal tissues, separate malignancy from pre-malignant pulmonary epithelium, and predict the prognosis of lung cancer patients [69-71]. Non-invasive approaches including analyzing proteins profiling of saliva and exhaled breath condensate have been promising in detecting lung cancer with AUC up to 0.90 [72, 73]. MS-based proteomic strategy is not only used in diagnosing lung cancer, further it can also be used in predicting the response to target therapy for lung cancer. For the majority of patients with advanced lung cancer, the most important biosignature is in predicting response to target treatment to achieve the goal of “personalized therapy”. Taguchi *et al*. developed MALDI MS algorithm to predict prognosis of non-small cell lung cancer patients after treatment with epidermal growth factor receptor tyrosine kinase inhibitors, which may help in the pretreatment selection of appropriate subgroups of lung cancer patients [74]. Although the results seem promising, these proteomic strategies remain investigational and await future validation of the application in screen, diagnosis, and pre-treatment selection of patients of lung cancer before they can be carried out in clinical practice.

**Table 1:** Selected representative studies of Mass spectrometry-based proteomics in Chest Medicine.

Disease	Specimen	Marker	Proteomic technique	Ref
Lung cancer	Tissues airway epithelium Saliva Tissues	Proteins profiling Proteins profiling Calprotectin, annexin A1, haptoglobin hp2, α 2-glycoprotein protein profiling, 17250 Da (-)	MALDI-TOF MALDI-TOF 2D-MS SELDI-TOF	[69] [70] [72] [71]
Lung cancer treatment response	Serum	Predictive algorithm	MALDI-TOF	[74]
COPD	Tissues bronchoalveolar lavage fluids Sputum Sputum	matrix metalloproteinase -13 and thioredoxin-like 2 neutrophil defensins 1 and 2, S100A8 (calgranulin A), and S100A9 (calgranulin B) 203 distinct proteins, protein profilings polymeric immunoglobulin receptor	MALDI-TOF SELDI-TOF CapLC-Q/TOF-MS 2D-DIGE-MS	[75] [76] [77] [78]
Asthma	exhaustion air exhaled breath Urine	Leukotrienes (LT) D4, LTE(4), LTB(4) LTB(4) LTE4	GC-MS LC-MS LC-MS	[79] [80] [81]

MALDI-TOF: matrix-assisted laser desorption inoization-time of flight; SELDI: Surface-enhanced laser desorption/ionization; 2D: two dimensional gel electrophoresis; DIGE: difference gel electrophoresis; LC: liquid chromatography; GC: gas chromatography; Q: quadrupole.

### 5.2. Aging and Gerontology

The study of elderly people whose age is more than 65 years is termed as geriatrics. In addition to the chronological definition, aging could still be defined biologically, physically and mentally. Biological aging represents a fundamental process that has a higher risk in the development of cancer, neurodegenerative, and cardiovascular diseases (CAD) than non-elderly [88].

With increasing longevity and decreased fertility rate, the elderly population is getting steadily increased worldwide. Agerelated chronic diseases, termed comorbidity and multimorbidity started to catch clinicians’ attentions [89]. CAD, Alzheimer’s disease, and cancer can be considered as accumulating disease predominantly observed in the aging period. Proteomics approach can reveal the phenotype of aging and may provide an insight for investigating the mechanism of these chronic diseases. Some important studies related to aging disease are listed in Table 2 [90-108].

MS can examine chemical structure and organizing process of amyloid beta-protein from Alzheimer’s brain [95, 109]. In addition to apply MS-based techniques in probing etiology and mechanism of Alzheimer’s disease, more studies have adopted MS-based proteomics for biomarker discovery of Alzheimer’s disease. Cerebral spinal fluid and serum have been the material for MS-based non-target proteomics and target-proteomics for discovering and validating biomarkers of Alzheimer’s disease, respectively [96, 97]. Likewise, MS-based proteomics lent insight into the pathogenic role of deregulated protein in pathophysiology of Alzheimer’s disease, which is helpful as a treatment target for drug discovery [97-99, 110]. In addition to effects of aging on developing disease, it is also observed that age had similar detrimental influence on proteins. Age-related modification (phosphorylation, oxidation, glycation, racemization, nitration, etc.) are also observed and may induce disease [106, 107].

### 5.3. Nephrology

Some MS-based proteomic studies encompassing ischemic acute kidney injury, contrast nephropathy, urolithiasis, kidney rejection, and lupus nephritis were also listed in Table 3 [69, 111-130]. The gold standard of diagnosing glomerulonephritis (GN) is renal biopsy, which is invasive and risky. MS-based proteomic studies have uncovered new biomarkers and pathophysiology of GN. Beck *et al*. have used MS approaches for renal tissue specimen analysis from patients with idiopathic membrane nephropathy. to identify M-type phospholipase A2 receptor, as a potential marker that differentiates patient groups between idiopathic membrane nephropathy and other GN [122]. A pattern consisting of 22 polypeptides from a capillary electrophoresis-mass spectrometry (CEMS) study has successfully distinguished IgA nephropathy from healthy controls, diabetic nephropathy, minimal change disease, and focal segmental glomerulosclerosis with 100% sensitivity [131]. There have been proteomic studies on peritoneal dialysate from patients receiving peritoneal dialysis [132]. It is believed that proteomics of peritoneal dialysate can enhance understanding of peritoneal dialysis and lend potential biomarkers for predicting peritoneal damage [128].

### 5.4. Omics-based personalized medicine: an evolving art of clinical practice

The revolution of medicine has entered a new era, with major achievements in recent decades. The challenging progress is the eager to pursue personalized medicine. Personalized medicine, meaning to take into consideration the whole system biologic status of an individual enables the public health scientists and clinicians to choose and tailor the appropriate screening strategy, intervention, drugs to fit the need of biological variability of each individual as possible [133]. Certainly, considering the heterogeneity of genome, epigenome, and the resulting associated phenotype, it is unlikely and cost to design a specific examination or create a medication just unique to one patient. American officials have defined personalized medicine with greater precision as “ability to classify individuals into subpopulations that differ in their susceptibility to a particular disease or their response to a specific treatment” [134].

**Table 2:** Selected representative studies of mass spectrometry-based proteomics in Aging and Gerontology.

Disease	Specimen	Marker	Proteomic technique	Ref
CAD	Plaque atherosclerotic plaques Platelet Blood	SDF1-α, unprocessed TGF-β1, basic FGF, PDGF Protein expression map Secretogranin III, cyclophilin A, and calumenin vimentin, mannose binding lectin receptor protein, S100A8 calcium-binding protein	LC-MS MALDI-TOF 2D-MALDI-TOF 2D-MALDI-TOF	[92] [90] [93] [140]
Alzheimer’s disease	cerebral Cortex cerebrospinal fluid Serum Leukocyte	amyloid β-protein unknown 7.7 kDa polypeptide, 4.8 kDa VGF polypeptide, cystatin C, two beta-2-microglobulin plasma ApoE levels had no obvious clinical significance 14-3-3 protein epsilon and peroxiredoxin 2; and eight downregulated proteins, actin-interacting protein, mitogen activated protein kinase 1, beta actin, annexin A1, glyceraldehyde 3-phosphate dehydrogenase, transforming protein RhoA, acidic leucine-rich nuclear phosphoprotein 32 family member B,	HPLC-MS SELDI-TOF HPLC-QTRAP MALDI	[95] [96] [97] [99]
Cancer	Urine from prostate cancer Urine from urothelial cancer	Polypeptides Polypeptides pattern	CE-MS CE-MS	[102] [141]
PTM	Lens	N-terminal racemization.	LC-MS/MS	[107]

PDGF: pigment epithelium-derived factor; VGF: vessel growth factor; SDF1-α: Stromal cell-derived factor α; TGF-β: Transforming growth factor-β1; CE-MS: capillary-electrophoresis-coupled mass spectrometry; PTM: posttranslational modification

**Table 3:** Selected representative studies of mass spectrometry-based proteomics in Nephrology.

Disease	Specimen	Marker	Proteomic technique	Ref
AKI	Urine Urine Urine	IP-10 Angiotensinogen Protein profiling	SELDI-TOF 2-D LC-MS/MS SELDI-TOF	[115] [116] [118]
GN	Urine kidney tissues kidney tissues serum	isoforms of hepcidin, fragments of alpha1-antitrypsin and albumin C3α and C3β Ig heavy chain amyloid. M-Type Phospholipase A2 Receptor	SELDI-TOF LC-MS LMD/MS LC-MS	[119] [120] [121] [122]
CKD	Urine Urine Urine	Peptide profiling urinary proteome-based classifier (CKD273) 12-peak proteomic signature	CE-MS CE-MS SELDI-TOF	[125] [126] [127]
Dialysis	Peritoneal dialysate Dialysate Dialysate	Protein profiling Protein profiling Protein profiling	nano LC-MS/MS SELDI-TOF nano-UPLC-MS/MS	[129] [130] [132]

AKI: acute kidney injury; GN: glomerulonephritis; IP-10: interferon-inducible protein-10; LC: liquid chromatography; LMD: laser microdissection; CE-MS: capillary-electrophoresis-coupled mass spectrometry.

In the past, universal personalized medicine seems impossible to carry out either in Western or Chinese clinical practice. In Western medicine, what most time physician spent in clinical practice is disease recognition and decision making. Physicians are trained to cure disease regardless of biological variance among individuals. Conceptually different, traditional Chinese medicine considered ill individual as a whole, thought of system medicine, and aimed to achieve system balance based on the concepts of yin–yang, Qi and Blood, and Zang-fu organ [135, 136]. However, traditional Chinese medicine could not be carried out and quantified uniformly by each practitioner, since the practice of Chinese medicine depends largely on imagery, intuitional, and holistic thinking [137].

Completion of Human genome project allows illumination of the human genome and eager in maturing of personalized medicine to resolve irreconcilable differences of philosophies between Western medicine and Chinese medicine [138]. Despite the availability of complete genome sequence, researchers cannot predict manifestation of diseases of physiological process very precisely, given expression of organism activity is much closer to level of functional genome rather than that of genome. Awareness of dynamic complexity of biological activity within human body lead personalized medicine moving beyond genomics, epigenomics, transcriptomics, and finally proteomics to get direct levels of functional insight. Chen *et al*. studied the proteome of individual colorectal cancer tissues of each patient and used it to establish a pilot model of MS-based proteomics in personalized medicine [139]. This study offers a roadmap for future related studies of personalized medicine; MS-based proteomics of personalized medicine, a key strategy to reform healthcare, is still in its infancy. Issues in clinical aspects of personalized medicine merit attention: well-controlled study for subgrouping; cut-off value and threshold of biomarkers for disease detection and treatment response variant among persons; effects of environment, genetics, and disease variability in a population. Proteotype within the organism is dynamic and varies with time. How to summarize and signify results of this dynamic proteome across samples and individuals poses a challenge in near future.

## 6. Acknowledgments

This work was funded by grants from the National Science Council, chronic kidney disease (BM102021124), diabetes (BM102010130) and stroke biosignature (BM10 2021169) projects from Academia Sinica, Taiwan.
